# Focused Attention Meditation as a Pre-Exercise Strategy for Reducing Anxiety in Speed Skaters

**DOI:** 10.3390/s26020475

**Published:** 2026-01-11

**Authors:** Yosuke Tomita, Mari Yokoo, Kaori Shimoda, Tomoki Iizuka, Eikichi Sakamoto, Koichi Irisawa, Fusae Tozato, Kenji Tsuchiya

**Affiliations:** 1 Department of Physical Therapy, Faculty of Health Care, Takasaki University of Health and Welfare, Takasaki 370-0033, Gunma, Japan; 2132049@takasaki-u.ac.jp (M.Y.); 1930201@takasaki-u.ac.jp (T.I.); irisawa@takasaki-u.ac.jp (K.I.); 2 Department of Rehabilitation Sciences, Graduate School of Health Sciences, Gunma University, Maebashi 371-8514, Gunma, Japan; 3 Department of Rehabilitation, Kurosawa Hospital, Takasaki 370-0033, Gunma, Japan; 4 Takasaki University of Health and Welfare Clinic, Takasaki 370-0033, Gunma, Japan; sakamoto-e@clinic.takasaki-u.ac.jp; 5 Department of Rehabilitation, Sendai Seiyogakuin University, Sendai 984-0022, Miyagi, Japan; fusaetozato@gmail.com; 6 Department of Rehabilitation, Nagano University of Health and Medicine, Nagano 381-2227, Nagano, Japan; tsuchiya.kenji@shitoku.ac.jp

**Keywords:** focused attention meditation, anxiety, high-intensity intermittent training, exercise physiology, heart rate variability

## Abstract

**Highlights:**

**What are the main findings?**

- A single 10-min session of focused attention meditation significantly reduced pre-exercise state anxiety in competitive speed skaters compared with random thinking and controlled breathing.
- Focused attention meditation reduced pre-exercise anxiety, whereas controlled breathing primarily modulated heart rate variability, and neither intervention affected HIIT performance.

**What are the implications of the main findings?**

- Brief focused attention meditation may be considered as a feasible and practical pre-exercise mental strategy for athletes preparing for high-intensity training.
- Incorporating meditation into routine training sessions may support psychological stability and autonomic regulation without compromising physical performance of the athletes.

**Abstract:**

Anxiety is a common psychological challenge among athletes, particularly in response to intense training sessions. This randomized crossover study investigated the immediate effects of a single session of focused attention meditation on anxiety, autonomic responses, and performance during high-intensity intermittent training (HIIT) in twenty-six university-level speed skaters. Participants completed three pre-exercise interventions (focused attention meditation, controlled breathing, and random thinking) on separate occasions in a randomized order. Following each intervention, participants performed a leg cycling-based HIIT protocol consisting of 20 s of maximal effort work followed by 10 s of passive rest, repeated for 8 sets using a cycling ergometer. State anxiety was assessed using the State–Trait Anxiety Inventory, and mood disturbance was evaluated using the Profile of Mood States. Autonomic and physiological responses were assessed via heart rate variability (coefficient of variation), oxygen uptake, and power output, measured before and after the intervention and the HIIT bout. Focused attention meditation significantly reduced state anxiety compared with random thinking (ΔSTAI: −5.0 [6.0] vs. −1.0 [4.3]; *p* < 0.05, effect size = 0.527), whereas controlled breathing primarily influenced heart rate variability (CV: 0.10 [0.11] vs. 0.07 [0.03]; *p* = 0.041, effect size = 0.736). No significant differences were observed among conditions in mean power output or fatigue index during HIIT. These findings suggest that single-session focused attention meditation may serve as a practical pre-exercise strategy for an immediate reduction in state anxiety, without compromising subsequent high-intensity exercise performance.

## 1. Introduction

Anxiety is a prevalent psychological factor among athletes that arises from various sources, including injuries, competitive pressure, and intensive training demands [1,2,3]. In recent years, reports of mental health crises among athletes have increased, highlighting the urgent need for psychological support in sports settings. These challenges not only affect well-being but also impair athletic performance, particularly during high-stakes competitions or physically demanding training [4]. For athletes engaged in strenuous training, such as high-intensity intermittent training (HIIT), pre-exercise anxiety may interfere with training performance and long-term adherence [5]. In addition, HIIT elicits immediate changes in mood and stress-related physiological responses following a single exercise bout [6]. Therefore, the integration of psychological interventions into physical training routines is increasingly recognized as essential for supporting both the mental and physical aspects of athletic development [7].

Among the available psychological interventions, meditation and controlled breathing have emerged as accessible and effective strategies for reducing anxiety [8,9,10]. Although both interventions focus on breathing, they differ fundamentally in their objectives, methods and underlying mechanisms. Focused attention meditation encourages present-moment awareness through passive observation of natural breathing combined with structured cognitive monitoring, such as repeated breath counting from one to ten [8]. This practice involves active cognitive engagement: participants must detect counting errors (e.g., skipping numbers or losing synchronization) and reset their attention to their breath, thereby training attentional control and error monitoring [11]. Importantly, participants do not attempt to control their breathing rate or depth; instead, they observe their natural respiratory rhythm as it occurs. This approach has been shown to improve emotional regulation and attentional control in athletes [9], potentially through engagement of prefrontal cortical networks involved in top-down executive function [11].

In contrast, controlled breathing techniques, such as slow-paced respiration at a fixed frequency, require the active manipulation of respiratory parameters to achieve specific autonomic effects [10]. Participants are instructed to breathe at a predetermined rate (e.g., 0.1 Hz, or 6 breaths per minute) with controlled inspiration-to-expiration ratios, typically using external pacing devices such as metronomes [12,13]. Unlike meditation, controlled breathing does not involve cognitive counting or error monitoring; the primary focus is on maintaining a prescribed respiratory pattern. This intervention can promote parasympathetic nervous system activity and reduce the physiological arousal associated with anxiety through bottom-up mechanisms such as enhanced respiratory sinus arrhythmia and increased vagal tone [10,12,13].

Despite the growing use of meditation and controlled breathing across clinical, educational, and athletic populations, empirical evidence directly comparing their immediate psychological and physiological effects before intense exercise remains limited. Previous studies have demonstrated that even brief sessions of focused attention meditation can reduce state anxiety and modulate autonomic activity [14,15,16], whereas controlled breathing—particularly at slow, paced frequencies—robustly influences autonomic regulation via respiratory–cardiac coupling [17,18]. However, because these approaches are thought to operate through distinct mechanisms, their differential effects on the pre-exercise mental state, autonomic dynamics, and subsequent physical performance have not been clearly delineated. In particular, little is known about how brief mental interventions influence immediate responses to high-intensity interval training (HIIT), which is characterized by substantial physiological and psychological demands.

To address this gap, the present study aimed to estimate the immediate effects of a brief focused attention meditation on anxiety, autonomic nervous system activity, and performance during HIIT, and to compare these effects with those of controlled breathing and a control condition involving random thinking. We hypothesized that focused attention meditation would induce changes in autonomic nervous system dynamics while simultaneously reducing pre-exercise anxiety, as suggested by prior evidence demonstrating reductions in State–Trait Anxiety Inventory (STAI) scores and improvements in mood following brief meditation [19,20,21]. In contrast, we hypothesized that controlled breathing primarily modulates autonomic activity, with comparatively limited effects on anxiety reduction [17,21,22]. Furthermore, given the mixed and limited evidence for the immediate effects of meditation on physical capacity [9,23], we hypothesized that neither intervention would result in significant changes in high-intensity intermittent exercise performance.

## 2. Materials and Methods

### 2.1. Participants

Twenty-six healthy young athletes from a university speed skating team participated in this study (Table 1). All participants were categorized as Tier 3 according to the athlete classification framework proposed by McKay et al. [24]. This tier corresponds to “highly trained/national level” athletes who engage in structured training programs (typically ≥ 5 sessions per week) and compete at the national level. All participants had finished within the top 8 in at least one national speed skating competition, and some had also participated in international competitions. All participants had previous experience with the HIIT performed in this study and none had prior experience with any type of meditation. Participants were excluded from the study if they reported musculoskeletal injuries that would limit their ability to perform cycling exercises on the upper or lower limbs. The participants were instructed to refrain from consuming caffeinated food and drinks, drugs, alcohol, tobacco, or any form of nicotine use within 24 h prior to the experiment and were also advised to refrain from taking anti-inflammatory or analgesic medications during this period. The participants were instructed not to train for 24 h before the experiment. Participants were instructed to maintain their normal training regimens and dietary habits throughout the study period, except for the specified 24-h pre-session restrictions. All participants followed a standardized team training program consisting of resistance training (two sessions/week), high-intensity interval training (three sessions/week), repetition training (two sessions/week), and technical practice (six sessions/week). However, no specific control was imposed on training activities occurring more than 24 h before each experimental session [25], as strict regulation of training schedules was not feasible in this competitive athlete population [26]. Compliance with the pre-session instructions was carefully monitored to ensure methodological rigor. All participants received detailed written and oral explanations of the experimental requirements during a pre-study team meeting, including the 24-h training restriction and dietary/substance limitations. Experimental sessions were individually scheduled to coincide with each athlete’s designated rest days, thereby minimizing both the risk of non-compliance and disruption to their ongoing training programs. Participants received reminder messages 24-48 h before each visit reiterating all requirements. On the day of each session, verbal confirmation of compliance was obtained from each participant before commencing any measurement. Participants were asked to confirm that they had (1) refrained from training for ≥24 h, (2) avoided caffeine within 24 h, (3) not consumed alcohol, tobacco, or medications, and (4) followed all other study requirements [16]. Only after confirming full compliance did we proceed with the experimental protocol. After explaining the study procedures and the risks involved, written consent was obtained from all participants. This study was approved by the Institutional Review Board (approval No. 2157).

### 2.2. Measures

#### 2.2.1. Primary Outcomes

**Figure 1:**
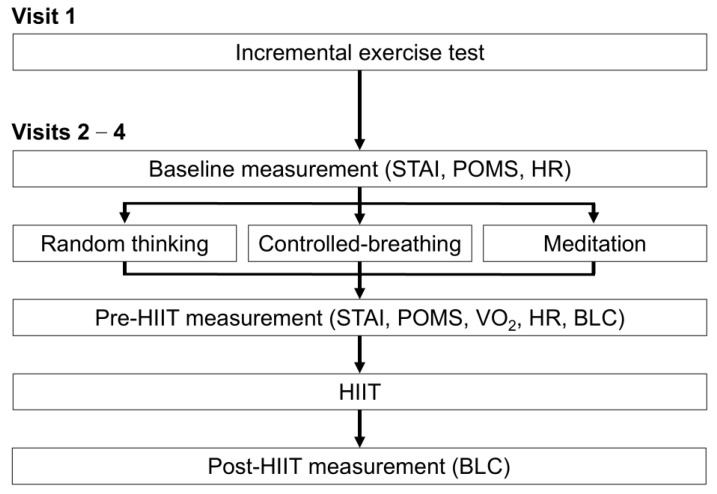
Experimental protocol.

This study had two primary outcomes: psychological state and power output measurements. Anxiety was assessed using the State–Trait Anxiety Inventory (STAI). The STAI comprises 40 items, with 20 allocated to each of the state and trait anxiety subscales. In the present study, only the state anxiety scale was used to capture changes in anxiety associated with the experimental intervention. State anxiety scores ranged from 20 to 80, with scores of 20–37 indicating low anxiety, 38–44 moderate, and ≥45 high anxiety, respectively [27]. Emotional regulation was evaluated using the total mood disturbance (TMD) score of the Profile of Mood States (POMS) [28], a widely used questionnaire for assessing transient mood states across six dimensions: tension–anxiety, depression–dejection, anger–hostility, vigor–activity, fatigue–inertia, and confusion–bewilderment. The TMD score was calculated by summing the negative mood subscales and subtracting the vigor score, providing a global index of mood disturbance. In athletic populations, POMS-TMD scores ranging from 0 to 40 are generally considered within the normal range, whereas scores above 50 indicate clinically meaningful mood disturbances [29]. The STAI and POMS questionnaires were administered immediately before and after each intervention condition (Figure 1). Changes in state anxiety and mood disturbance were calculated as the difference between the post- and pre-intervention scores and are reported as ΔSTAI state and ΔPOMS TMD, respectively.

Power outputs, including the mean power and fatigue index [30] during HIIT, were used as primary outcomes. FI was calculated as follows:

$$FI\left(\%\right)=\frac{\left(peak\;power\;\left(W\right)-minimum\;power\;(W)\right)}{peak\;power\;(W)}\times 100$$

#### 2.2.2. Secondary Outcomes

Blood lactate concentration, heart rate, heart rate variability (HRV; coefficient of variation [CV] of heart rate), and oxygen uptake were compared between the two interventions. Heart rate and oxygen uptake were expressed as % V̇O_2_max.

### 2.3. Design and Procedures

The experimental procedure is shown in Figure 1. Each participant visited the laboratory four times, with an interval of ≥1 week between sessions. During the first visit, an incremental exercise test was performed using a leg-cycling ergometer (Powermax-V3; Konami, Tokyo, Japan). All measurements and interventions were performed in the same quiet room, where the room temperature was maintained at 23 °C.

During the three subsequent visits, the participants performed HIIT under different experimental conditions (Figure 1). The order of the experimental conditions was randomized to minimize confounding effects. The participants were connected to the expired gas analysis system during all experimental conditions to ensure consistency in physiological measurements and avoid potential confounding effects related to device attachment. The duration of the pre-exercise intervention in the present study was set at 10 min based on previous studies demonstrating that short focused attention meditation and paced breathing interventions (approximately 5–10 min) can elicit immediate changes in psychological states and autonomic nervous system activity, even in meditation novices [14,15,16]. In addition, a 10-min duration was selected to ensure practical feasibility as a pre-exercise strategy that can be implemented immediately before high-intensity training without disrupting athletes’ regular routines. After completing the experimental conditions, HIIT was performed following the Tabata protocol [31], in which the participants repeated maximal sprint cycling for 20 s with a 10 s interval between them. Blood lactate concentration (BLC), heart rate, oxygen uptake, and psychological state were measured. BLC was measured using a lactate analyzer (Lactate Pro 2; Arkray, Kyoto, Japan). Arterial blood samples (0.3 μL) were obtained from the ears. The first blood drop was removed to avoid contamination of the samples. The heart rate was measured using a wearable electrocardiogram sensing device (MyBeat; Union Tool Co., Tokyo, Japan). The device was worn around the epigastrium on the participant’s chest, and the peak of the QRS complex was used to obtain the RRI time series. Oxygen uptake was measured using an electric gas flow meter (AE100i; Minato Medical Science, Osaka, Japan).

The participants underwent an initial visit for incremental exercise testing. During Visits 2–4, the participants first completed baseline measurements, including the State–Trait Anxiety Inventory (STAI), Profile of Mood States (POMS), heart rate [HR], and blood lactate concentration (BLC). Participants then completed randomized pre-exercise interventions (focused attention meditation, controlled breathing, or random thinking), followed by pre-high-intensity intermittent training (HIIT) assessments (STAI, POMS, oxygen uptake [V̇O_2_], HR, and BLC). Subsequently, the participants performed HIIT following the Tabata protocol at an exercise load of 160% V̇O_2_max. Post-HIIT BLC was measured 3 min after exercise. Each session was separated by at least one week.

#### 2.3.1. Incremental Exercise Test (V̇O_2_max)

An incremental exercise test was performed to determine the HIIT workload with minor modifications to the test protocol from previous studies. After a 2-min warm-up with no load, the participants initiated the test at a work rate of 70 W, which was increased by 35 W every 2 min, and the pedaling cadence was maintained at 70 rpm. The test continued until the participants could no longer maintain a pedaling rate of 70 rpm for 5 s or reported fatigue [32]. The participants wore a mask that covered their nose and mouth, to which a flow sensor and gas sampling tube were connected. The expired volume and gases were continuously analyzed using an electric gas flow meter (AE100i; Minato Medical Science, Osaka, Japan). All participants met at least one of the predefined criteria for V̇O_2_max determination, including achieving ≥90% of the age-predicted maximal heart rate, exhibiting a plateau in oxygen uptake (ΔV̇O_2_ < 150 mL·min^−1^), or attaining a respiratory exchange ratio > 1.1. No participants were excluded due to failure to meet these criteria. Verbal encouragement was provided during the tests.

#### 2.3.2. High-Intensity Intermittent Training (HIIT)

The HIIT included a 20-s exercise performed using a leg cycling ergometer (Powermax-V3; Konami, Tokyo, Japan) at an intensity of 170% of the participant’s V̇O_2_max, following the Tabata protocol [31]. Each bout was separated by a 10-s passive rest on the ergometer, and the procedure was repeated eight times for each participant. Expired gas (AE100i; Minato Medical Science, Osaka, Japan) and heart rate (myBeat; Union Tool Co., Tokyo, Japan) were continuously collected during the exercise and rest periods to measure oxygen uptake. BLC was measured using a lactate analyzer (Lactate Pro 2; Arkray, Kyoto, Japan) before and 3 min after the HIIT.

#### 2.3.3. Meditation

Participants performed su-soku, a form of focused attention meditation, for 10 min while seated on a chair with a backrest [33]. The participants were instructed to mentally count each complete breath cycle, defined as one inhalation followed by one exhalation, as a single unit. The participants continuously counted their breath cycles from one to ten, restarted from one cycle, and repeated the process throughout the session. They were instructed to keep their eyes closed and maintain attention to their breath, despite the presence of distracting thoughts. In cases where they made a counting error (e.g., repeating or skipping a number, forgetting to count a cycle, or failing to synchronize counting with actual breathing), they were instructed to reset the count and simultaneously press a handheld counter using their right thumb to monitor their attentional lapses [34,35].

#### 2.3.4. Controlled Breathing

Controlled breathing was performed based on previous studies at 0.1 Hz (one respiration cycle per 10 s), where the ratio of inspiration and exhalation was controlled to be 3:7 using a metronome for 10 min [10,36]. The participants were instructed to sit on a chair and adjust their breathing according to a metronome with their eyes closed. The accuracy of the respiration cycle was verified using the respiration rate acquired with an expired gas monitoring device (AE100i; Minato Medical Science, Osaka, Japan).

#### 2.3.5. Control Condition: Random Thinking

The random thinking intervention was based on a previous study [37]. The control intervention was performed for 10 min. Participants were instructed to sit on a chair and listen to randomly recorded conversations or local radio advertisements via speakers to allow their thoughts to come to mind without falling asleep. This intervention encourages random thinking because the recorded content is not associated with each other [37]. A researcher evaluated the participants’ performance from behind to check for unrelated tasks during the intervention, such as physical movements or sleeping.

Heart rate variability was calculated from the R–R interval data recorded during the intervention period for each experimental condition (focused attention meditation, controlled breathing, and random thinking). These data segments were selected to capture the autonomic nervous system activity during the respective pre-HIIT interventions. Oxygen uptake (V̇O_2_) and heart rate were analyzed using data collected continuously during the HIIT session to evaluate the physiological responses to high-intensity exercise.

### 2.4. Statistical Analysis

The sample size was determined based on the effect size of meditation on anxiety among college students [19]. A reported effect size of 0.554 and statistical power of 0.8 were used, and a total of 20 participants were required. Non-parametric statistical tests were adopted in this study because the Shapiro–Wilk test indicated that several outcome variables did not follow normal distribution. Accordingly, all continuous data, including participant characteristics, are presented as median and interquartile range (IQR) to ensure consistency between data presentation and statistical analysis. For each outcome, Friedman’s test was first applied as a non-parametric omnibus test to assess the overall differences across the three experimental conditions. When the Friedman test reached statistical significance, post-hoc pairwise comparisons were conducted using Wilcoxon signed-rank tests with Bonferroni correction. Effect sizes (r) for post-hoc pairwise comparisons were calculated as r = Z/√N from the Wilcoxon signed-rank test and interpreted as small (0.10), medium (0.30), and large (≥0.50) [38,39]. The statistical significance level was set at 5%. SPSS version 21 (IBM, Japan, Tokyo) was used for statistical analysis.

## 3. Results

**Figure 2:**
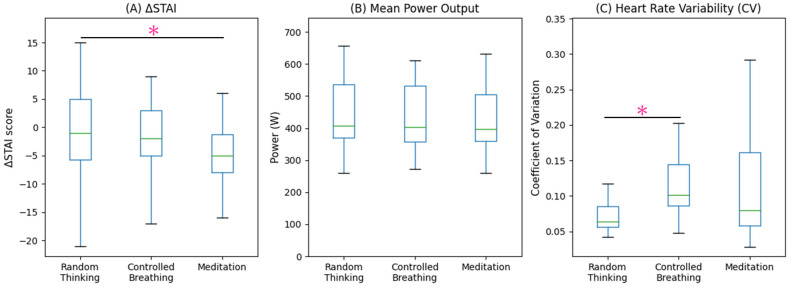
Box plots illustrating differences in key outcomes across the three experimental conditions. (**A**) change in state anxiety score (ΔSTAI), (**B**) mean power output during high-intensity intermittent training (HIIT), and (**C**) heart rate variability during the intervention period. * *p* < 0.05.

All 26 participants completed the intervention and HIIT protocols without adverse events. The baseline STAI trait and state scores were 41.0 (14.0) and 42.0 (14.5), respectively. The baseline POMS-TMD score was 30.0 (18.5). The primary and secondary outcome measures are shown in Table 2 and Figure 2.

Significant differences were observed in the changes in state anxiety scores (ΔSTAI state) across the three interventions (χ^2^ = 10.469, *p* = 0.026). Post-hoc comparisons with Bonferroni correction revealed that focused attention meditation resulted in a significantly greater reduction in state anxiety than random thinking (median difference: −3.0; 95% confidence interval: −5.5 to −1.0; *p* = 0.018, effect size r = 0.527, power = 0.863). No significant differences were observed between the controlled breathing and random thinking conditions or between the meditation and controlled breathing conditions.

A significant intervention effect was observed on the coefficient of variation (CV) of the heart rate (χ^2^ = 6.381, *p* = 0.041). Post-hoc analysis showed that CV was significantly greater after controlled breathing than after random thinking (median difference: 0.03; 95% confidence interval: 0.019 to 0.094; *p* < 0.05, effect size r = 0.736, power = 0.998). No significant differences in CV were observed between the focused attention meditation and controlled breathing or between the focused attention meditation and random thinking groups.

No significant differences were observed among the three interventions in any performance or physiological outcome during HIIT, including mean power output, fatigue index, postexercise blood lactate concentration, peak heart rate, time to reach peak heart rate, peak V̇O_2_, and time to peak V̇O_2_ (all *p* > 0.05).

## 4. Discussion

The results showed that focused attention meditation significantly reduced state anxiety compared with the control condition, whereas controlled breathing did not differ significantly from the control. No significant difference in anxiety reduction was observed between focused attention meditation and controlled breathing. Furthermore, neither intervention affected physical performance during HIIT. With respect to autonomic outcomes, heart rate variability was significantly increased during controlled breathing compared with the control condition, whereas no significant difference was observed between focused attention meditation and the control. In addition, heart rate variability did not differ significantly between controlled breathing and focused attention meditation. Taken together, these findings suggest that focused attention meditation and controlled breathing may serve distinct roles through different underlying mechanisms. Focused attention meditation may be more closely related to cognitive–emotional processes involved in pre-exercise anxiety, whereas controlled breathing may primarily act on physiological regulation, effects that may not necessarily be reflected in state anxiety measures.

Athletes frequently encounter mental and psychological challenges, including elevated anxiety levels, which can negatively affect their performance and overall well-being [40]. Therefore, maintaining and promoting mental health throughout an athlete’s career is critical, underscoring the importance of integrating mental and physical interventions into routine training [7,41]. HIIT has been widely recognized for its efficacy in improving both aerobic and anaerobic performance across diverse athletic [34,42] and clinical populations [43,44,45]. However, the demanding nature of HIIT imposes considerable psychological stress, which may adversely affect athletes’ mood state. Previous research has shown that repeated exposure to HIIT sessions is associated with increased negative mood components, such as fatigue and tension, along with an overall disturbance of mood profiles in athletes [46]. Our findings suggest that a brief 10-min session of focused attention meditation can immediately reduce state anxiety and modulate autonomic nervous system activity prior to HIIT, supporting the value of pre-exercise mental regulation strategies in reducing anxiety.

Regarding the magnitude of the observed effects, the reductions in state anxiety were statistically significant but modest in size. Nevertheless, even small changes in pre-exercise anxiety may be practically meaningful in high-intensity exercise contexts, where psychological readiness and perceived stress influence performance quality, exercise tolerance, and willingness to engage in demanding tasks [47,48,49]. Accordingly, interventions yielding modest anxiolytic effects should be considered relevant when implemented immediately before high-load exercise bouts, such as HIIT.

With respect to physiological responses, differences in heart rate variability, as reflected by the coefficient of variation in heart rate, were observed across conditions. Specifically, controlled breathing was associated with greater heart rate variability compared than random thinking, whereas focused attention meditation did not differ significantly from random thinking. Moreover, heart rate variability during meditation showed greater inter-individual variability, suggesting heterogeneous physiological responses. These findings indicate that intentional manipulation of breathing patterns can robustly modulate cardiac variability prior to exercise, whereas the autonomic effects of focused attention meditation appear to be less consistent across individuals. Controlled breathing strongly modulates cardiac variability via respiratory–cardiac coupling, thereby increasing the magnitude of heart rate variability through bottom-up physiological mechanisms [18,22]. This mechanism plausibly explains the greater heart rate variability observed during controlled breathing compared with random thinking in the present study. In contrast, focused attention meditation incorporates additional attentional and cognitive regulation processes, such as sustained attention and reduced engagement in ruminative or anticipatory thought patterns, which are central to reducing anxiety [50]. Unlike controlled breathing, focused attention meditation does not aim to directly manipulate respiratory parameters but instead promotes present-moment awareness through passive attention to breathing. Neurocognitive evidence suggests that this practice engages the prefrontal regulatory networks involved in top-down attentional control and emotional regulation [11,34]. These cognitive components may explain why meditation was associated with reductions in state anxiety, despite showing autonomic effects that were broadly comparable to those of controlled breathing in some indices.

The present findings extend previous research demonstrating the anxiolytic effects of mindfulness-based interventions in athletic populations [51,52] by showing that even a single brief session of focused attention meditation can be effective when implemented immediately before vigorous physical exercise. Importantly, this anxiolytic effect was observed without compromising physical performance during HIIT. Together, these findings provide empirical evidence that focused attention meditation may enhance mental readiness for forthcoming high-intensity training while preserving physical output. Beyond these outcome-level effects, the potential value of focused attention meditation lies in its underlying cognitive characteristics, rather than autonomic modulation alone. Unlike controlled breathing, which primarily targets physiological regulation through the explicit manipulation of respiratory parameters, focused attention meditation requires active cognitive engagement, including sustained attention, detection of attentional lapses, and deliberate redirection of focus to the present moment. These processes are central to anxiety regulation and may help attenuate anticipatory or ruminative thought patterns prior to exercise. From a practical perspective, focused attention meditation may be particularly advantageous for athletes with little or no prior meditation experience. Because it relies on simple and concrete instructions, such as attending to the natural rhythm of breathing and counting breaths, it can be readily implemented without extensive training or specialized equipment. This accessibility enhances the feasibility and potential adherence in applied sports settings, especially when brief interventions are required immediately before high-intensity training.

This study had several strengths that enhanced the validity and applicability of its findings. First, we adopted a comprehensive set of outcome measures spanning the psychological (STAI and POMS), physiological (V̇O_2_ and heart rate), and performance-related (power output) domains. This multidimensional assessment allowed us to characterize the distinct effects of focused attention meditation and paced breathing on psychological and physical readiness prior to HIIT. Second, the use of a randomized crossover design strengthened the internal validity of the study by controlling for the effects of individual variability and order. Each participant served as a control, which improved the statistical power and reduced the influence of confounding factors.

Despite these strengths, the present study had several limitations. First, adherence to the intervention was not objectively verified. In the focused attention meditation condition, compliance was inferred from participants’ self-reported counter presses without independent confirmation of breath-counting accuracy or attentional lapses. Similarly, adherence in the random thinking condition relied primarily on researcher observation rather than objective monitoring. Future studies should incorporate more rigorous methods to objectively assess compliance with the intervention protocols. Second, this study examined only the immediate effects of a single, brief (10-min) intervention session. Consequently, the long-term impact of regular meditation or breathing practice on anxiety and exercise performance remains unknown. In addition, because only one intervention duration was tested, the minimal effective dose required to elicit meaningful psychological or physiological changes could not be determined in this study. Therefore, future studies should examine different intervention durations to clarify potential dose–response relationships. Third, the present findings may have been influenced by substantial inter-individual variability. Participants differed in baseline anxiety levels prior to the interventions and in their prior familiarity with high-intensity intermittent training (HIIT), which may have attenuated anticipatory anxiety responses in some individuals. In addition, responses to focused attention meditation may vary considerably among individuals, particularly when the practice is new. Consistent with this notion, heart rate variability, expressed as the coefficient of variation, exhibited marked inter-individual variability, especially in the meditation condition. This heterogeneity may have contributed to the absence of consistent autonomic effects across participants. Fourth, owing to the limited sample size, this study focused on the total mood disturbance (TMD) score of the Profile of Mood States (POMS) without conducting formal analyses of individual subscales. Although exploratory analyses were performed, they were considered underpowered and therefore not interpreted, given the increased risk of type I error associated with multiple comparisons. Future studies with larger samples should investigate specific POMS subscales to provide a more detailed understanding of the psychological responses. Fifth, sex-stratified analyses were not performed because of insufficient statistical power in the present study. Future studies with larger cohorts are needed to explore potential sex-specific responses and interactions. Finally, all participants in this study were experienced athletes familiar with HIIT, which may have reduced their baseline anticipatory anxiety levels. Consequently, the anxiolytic effects of focused attention meditation observed in this study may be more pronounced in individuals with higher pre-exercise anxiety, such as novice exercisers or clinical populations. Further research is required to determine the generalizability of these findings across different populations and training contexts in the future.

## 5. Conclusions

This study examined the immediate effects of a single session of focused attention meditation on anxiety and performance during high-intensity intermittent training. The results showed that focused attention meditation was associated with a reduction in state anxiety compared with a control condition, while no changes in physical performance during HIIT were observed. In addition, autonomic responses during the intervention period differed across conditions, as reflected by changes in heart rate variability magnitude. These findings indicate that brief focused attention meditation may represent a feasible pre-exercise approach for supporting psychological readiness in athletes undergoing high-intensity training, without adversely affecting subsequent exercise performance.

## Figures and Tables

**Table 1:** Participant demographics (n = 26), median (interquartile range).

Age (years)	19.0 (1.8)
Sex (male/female)	12/14
Height (cm)	163.5 (12.0)
Body weight (kg)	60.3 (11.5)
Athlete classification	Tier 3 (national level)
Meditation experience	None

**Table 2:** Comparisons of outcome measures between interventions (n = 26).

	Random Thinking	Controlled Breathing	Meditation	χ^2^	*p*-Value	ES	Power
ΔSTAI state	–2.0 (11.0)	–2.0 (9.5)	–5.0 (10.0) *	10.469	0.026	0.527	0.863
ΔPOMS TMD	–4.0 (11.5)	–2.0 (5.5)	–3.0 (6.0)	0.433	0.805	-	-
Mean power (%V̇O_2_ max)	153.0 (14.7)	153.3 (13.5)	150.8 (15.8)	1.520	0.468	-	-
FI (%)	86.8 (11.6)	86.5 (15.2)	83.2 (0.595)	1.520	0.595	-	-
BLC after HIIT (mmol/L)	12.1 (5.0)	12.4 (4.1)	12.2 (5.9)	0.571	0.751	-	-
Peak heartrate (bpm)	181.6 (7.5)	182.1 (10.1)	182.0 (7.4)	0.095	0.953	-	-
Time to peak heartrate (s)	228.9 (59.0)	226.2 (50.5)	229.2 (31.6)	0.857	0.651	-	-
Peak V̇O_2_ (%V̇O_2_ max)	93.7 (13.8)	83.6 (14.1)	96.4 (9.8)	3.846	0.146	-	-
Time to peak V̇O_2_ (s)	231.0 (138.5)	223.0 (90.0)	203.0 (102.5)	1.385	0.500	-	-
CV of heartrate	0.07 (0.03)	0.10 (0.11) *	0.08 (0.18)	6.381	0.041	0.736	0.998

Data are presented as medians (interquartile ranges). * Significant difference with random thinking. † Significant difference with paced breathing. STAI, State–Trait Anxiety Inventory; POMS, Profile of Mood States; ΔSTAI state, difference in State–Trait Anxiety Inventory state score from baseline to post-intervention; FI, fatigue index; BLC, blood lactate concentration; HIIT, high-intensity intermittent interval training; CV, coefficient of variation; ES, effect size. Effect size and statistical power were estimated for the post-hoc test results of the two interventions.

## Data Availability

Data are available upon request.

## Abbreviations

The following abbreviations are used in this manuscript:

HIIT	High-Intensity Intermittent Training
STAI	State–Trait Anxiety Inventory
POMS	Profile of Mood States
TMD	Total Mood Disturbance
ΔSTAI	Change in State–Trait Anxiety Inventory State Score
ΔPOMS TMD	Change in Profile of Mood States Total Mood Disturbance
FI	Fatigue Index
BLC	Blood Lactate Concentration
HR	Heart Rate
HRV	Heart Rate Variability
RRI	R–R Interval
CV	Coefficient of Variation
V̇O_2_	Oxygen Uptake
V̇O_2_max	Maximal Oxygen Uptake
rpm	Rotations per Minute
ECG	Electrocardiogram
